# A cross-sectional study clarifying profiles of patients with diabetes who discontinued pharmacotherapy: reasons and consequences

**DOI:** 10.1186/s12902-021-00778-7

**Published:** 2021-06-14

**Authors:** Yoshiko Tominaga, Donald E. Morisky, Mayumi Mochizuki

**Affiliations:** 1 Social Pharmacy, Faculty of Pharmaceutical Sciences, Niigata University of Pharmacy and Applied Life Science, Niigata, Japan; 2 Division of Hospital Pharmacy Science, Faculty of Pharmacy, Keio University, Tokyo, Japan; 3 Department of Community Health Sciences, UCLA Fielding School of Public Health, Los Angeles, California United States of America

**Keywords:** diabetes, discontinuation, adherence, persistence, disease perception

## Abstract

**Background:**

Although diabetes is one of the fastest increasing diseases in prevalence worldwide and demands significant medical resources, more than half of all patients with diabetes do not achieve the expected target level of blood glucose. As a potential cause of poor glycemic control, insufficient adherence to medication has long been discussed and variably studied. However, dropout from treatment as another plausible cause has not been fully examined. The aim of this study was to clarify profiles of patients with diabetes who discontinued pharmacotherapy (Discont group) by extracting reasons of their decisions and by comparing with those who continued (Cont group) in terms of the related factors to glycemic control.

**Methods:**

A cross-sectional, internet-based survey was conducted among Japanese with diabetes registered in a database. A self-administered questionnaire consisting of the 8-item version of the Morisky Medication Adherence Scale (MMAS-8), glycosylated haemoglobin (HbA_1c_) level, and demographic and disease characteristics was completed by all participants. Reasons for medication discontinuation and resumption were also received retrospectively from participants in the Discont group. To examine the risk of uncontrolled HbA_1c_, logistic regression analysis was conducted in each group.

**Results:**

In the Discont group (148 cases), older age at resumption of pharmacotherapy and current smoking habit increased the probability of uncontrolled HbA_1c_, whereas in the Cont group (146 cases), a familial history of diabetes and better medication adherence in MMAS-8 scores decreased the probability of uncontrolled HbA1c. A relationship between medication adherence and HbA_1c_ level was seen in the Cont but not in the Discont group. About 70 % of those in the Discont group made their decision to terminate diabetes treatment without consulting physicians and half of them perceived their situations inappropriately.

**Conclusions:**

Those who discontinued pharmacotherapy were less adherent to medication than those who did not discontinue. Risk factors for glycemic control also differed between those who discontinued and those who did not. More than one-third of participants with diabetes who discontinued pharmacotherapy had inappropriate perceptions of their disease, which medical professionals should be aware of for better interventions.

## Introduction

Diabetes is one of the fastest increasing diseases in prevalence worldwide, and its prevalence was estimated to be 463 million individuals in 2019. Medical expenditure associated with diabetes accounted for $76 billion in 2019, approximately 10 % of the total [1]. Various types of potent oral or injectable medications are now available for daily practice. However, a Japanese study revealed that more than half of all patients with diabetes do not achieve the target level of blood glucose, below 7.0 % (52 mmol/mol), [2] which is recommended by the Japanese Diabetes Society [3]. One of the potential causes of poor glycemic control may be insufficient adherence to medication. According to the studies about medication adherence in diabetes treatment, depression and medical costs are found to be consistent factors in systematic review [4]. Also, age [5], self-efficacy [6], and personality traits [5] are potential factors to be associated with medication adherence. However, knowing underlining potential factors does not mean to solve nonadherence problems because multiple factors may exist in each individual patient and some of them are not modifiable.

In addition to adherence, persistence with medication is another critical issue. Some studies using administrative claims databases of relatively large samples presented specific rates of persistence [7, 8]. Comparative analysis across types of drugs were also reported [9] and could be applied in selecting medications to lower the risk of discontinuation. Those analyses reflected real-world circumstances but did NOT give reasons for discontinuation of prescribed pharmacotherapies.

An integrated guidance document suggested methods to improve attendance at scheduled appointments for diabetes care [10]. That document, based on various surveys and reports in the literature, cited reasons for dropouts from the Japan Diabetes Outcome Intervention Trial 2 (J-DOIT2) [11]. Its endpoint of discontinued appointments could be a valuable source for estimating medication persistence among the population with diabetes, although the consequences of discontinuation could not be tracked.

The primary purpose of this study was to investigate the specific characteristics of participants who discontinued pharmacotherapy for the treatment of diabetes in comparison with those who did not in right of the associated factors with glycemic control in each patient group. The secondary one was to quantify the reasons for discontinuation of diabetic pharmacotherapy.

## Participants and methods

### Participants and data collection

A cross-sectional, internet-based survey was conducted among individuals with diabetes registered (more than 7,000) in a Japanese research company database (Rakuten Insight Co., Ltd. https://insight.rakuten.co.jp/) which serve approximately 320,000 patents in total. Those with type 2 diabetes who were currently receiving pharmacotherapy were regarded as eligible and invited to enrol in this study. At enrolment, they were pre-screened for inclusion in either the population who had discontinued pharmacotherapy for more than six months (Discont group) or the population who had not discontinued (Cont group) before completing a self-administered questionnaire.

The first section of the questionnaire for both populations consisted of the 8-item version of Morisky Medication Adherence Scale (MMAS-8, Table 1), glycosylated haemoglobin (HbA_1c_) level, gender, age, body mass index, duration since diagnosis of diabetes (diabetes duration), diabetes medications (number of medications for diabetes), complications associated with diabetes (complications), experience of drug-related side effects, family history of diabetes and current smoking habit. The MMAS-8 was confirmed to be a reliable self-administered questionnaire for medication adherence [12–14]. The MMAS-8, which was developed in reference to psychometric properties, does not directly calculate the complying rate of taking medication but is confirmed to be consistent with the rate of taken pills. The second section of the questionnaire was completed only by those in the Discont group. It contained the period not receiving pharmacotherapy (discontinuation duration), discontinuation duration divided by diabetes duration (discontinuation ratio), and age when resuming pharmacotherapy (age at resumption).

**Table 1:** Morisky Medication Adherence Scale (MMAS-8) questionnaire^1^

Q1	Do you sometimes forget to take your diabetic medication(s)?
Q2	People sometimes miss taking their medications for reasons other than forgetting. Thinking over the past two weeks, were there any days when you did not take your diabetic medication(s)?
Q3	Have you ever cut back or stopped taking your medication(s) without telling your doctor, because you felt worse when you took it?
Q4	When you travel or leave home, do you sometimes forget to bring along your diabetic medication(s)?
Q5	Did you take your diabetic medication(s) yesterday?
Q6	When you feel like your blood glucose is under control, do you sometimes stop taking your medication(s)?
Q7	Taking medication(s) every day is a real inconvenience for some people. Do you ever feel hassled about sticking to your diabetic treatment plan?
Q8	How often do you have difficulty remembering to take all your medication(s)?

^1^MMAS-8: 8-item version of the Morisky Medication Adherence Scale. The MMAS (8-item) content, names and trademarks are protected by US copyright and trademark laws. Permission for use of the scale and its coding is required. A license agreement is available from: MMAR, LLC, Donald E. Morisky, ScD, ScM, MSPH, 294 Lindura Court, Las Vegas, NV 89,138 − 4632; USA; dmorisky@gmail.com

Participants in the Discont group were asked to choose among possible answers to the question: ‘Why did you stop pharmacotherapy?’ They were allowed to give more than one answer, and the potential responses provided were selected based on preceding studies [10, 15–17]. The number giving each possible response and percentages of respondents selecting each answer as well as percentages in each group, which were combined by eliminating overlapping counts in the same group, were calculated. Study participants in the Discont group were also asked the open-ended question: ‘What triggered your resumption of pharmacotherapy?’ to which they gave a free descriptive answer. Reasons for pharmacotherapy discontinuation and resumption were categorized by underlying commonalities.

Recruitment was completed during April 2017, when it was expected that the study population would reach approximately 150 in each group.

### Statistical analysis

The MMAS-8 was coded by a designated rule and calculated for total scores, which ranged from 0 to 8. The higher the total score, the better the medication adherence. According to the standard categorization of the MMAS-8, high (8), medium (< 8 and ≥ 6), and poor (< 6) adherents were determined. For the measurement of glycemic control, the submission of at least three separate glycosylated haemoglobin (HbA_1c_) test results within one year was requested to be averaged for analysis.

To examine the relationship between HbA_1c_ level and other variables in each population, HbA_1c_ was converted to binary data, i.e., ‘controlled’ (HbA_1c_ < target) or ‘uncontrolled’ (HbA_1c_ ≥ target). The target level was theoretically determined for each patient in reference to the treatment guidelines prepared by the Japan Diabetes Society [3]. Based on the guidelines, the target for HbA_1c_ control among those receiving medications (i.e., insulin, sulfonylurea and glinide agents) and at potential serious risk of hypoglycaemia aged ‘65 years or older and below 75 years’ or ‘75 years or older’ was assumed to be below 7.5 % (58 mmol/mol) or below 8.0 % (63 mmol/mol), respectively. The HbA_1c_ control target for all others was assumed to be below 7.0 % (52 mmol/mol). For logistic regression analysis with the dependent variable of HbA_1c_ level in each population, univariate analyses with variables collected as demographic and diseases characteristics were conducted beforehand. Then, the variables with p values of less than 0.2 were entered to multivariate logistic regression analysis. The odds ratio (OR) for the risk of HbA_1c_ ‘uncontrolled’ versus ‘controlled’, 95 % CI and p value of each dependent variable were calculated.

All p values of less than 0.05 on 2-sided tests were regarded as representing statistically significant differences. JMP 14 software (SAS Institute Inc., Cary, NC, USA) was used for all data analyses.

## Results

### Demographic and disease characteristics

A total of 294 eligible responses, 148 from the Discont and 146 from the Cont group, were enrolled. Descriptive statistics on the data collected for each population are presented in Table 2. Most variables were comparable in the two populations. The HbA_1c_ level was slightly higher in the Discont group (7.4 ± 1.4 %, 58.3 ± 16.5mmol; mean ± SD) than in the Cont group (7.2 ± 1.1 %, 55.1 ± 11.7mmol), but the difference did not reach statistical significance (*p* = 0.131). The proportion of participants with controlled HbA_1c_ was the same (53 %) in both groups. However, the medication adherence rate as determined by the three MMAS-8 levels (high, medium and low) differed significantly between the Discont (20 %, 39 and 41 %, respectively) and Cont (35 %, 30 and 36 %, respectively) groups.

**Table 2:** Demographic and disease characteristics of study participants

Variable			Discontinuation (*n* = 148)	Continuation (*n* = 146)	*p* value
Gender: male		Cases (%)	114 (77)	102 (70)	0.164^2^
Age (yr)		Mean ± SD	54.4 ± 10.1	54.6 ± 9.5	0.887^3^
Body mass index (%)		Mean ± SD	26.3 ± 5.6	26.8 ± 5.8	0.463^3^
Diabetes duration (yr)		Mean ± SD	12.2 ± 7.8	11.5 ± 7.8	0.454^3^
Discontinuation duration (yr)		Mean ± SD	2.6 ± 2.4	—	—
Discontinuation ratio (%)		Mean ± SD	23.1 ± 22.0	—	—
Age at resumption (yr)		Mean ± SD	50.9 ± 9.6	—	—
Complications		Cases (%)	22 (15)	15 (10)	0.235^2^
Number of medications^1^		Mean ± SD	4.2 ± 3.4	4.3 ± 3.9	0.791^3^
Insulin use		Cases (%)	24 (16)	21 (14)	0.608^2^
Hypoglycaemia (episodes per year)		Cases (%)	27 (18)	30 (21)	0.617^2^
Drug-related side effects		Cases (%)	34 (23)	34 (23)	0.949^2^
Familial history of diabetes		Cases (%)	75 (51)	62 (43)	0.158^2^
Current smoking habit		Cases (%)	27 (18)	31 (21)	0.520^2^
HbA_1c_ (%) (mmol/mol)		Mean ± SD	7.4 ± 1.4 58.3 ± 16.4	7.2 ± 1.1 55.1 ± 11.7	0.131^3^
HbA_1c_ under control		Cases (%)	79 (53)	78 (53)	0.994^2^
MMAS-8 adherence classification	High (8)	Cases (%)	30 (20)	51 (35)	0.017^2^
	Medium (≥ 6 and < 8)	Cases (%)	57 (39)	43 (30)	
	Low (< 6)	Cases (%)	61 (41)	52 (36)	

^1^Number of medication for diabetes ^2^Pearson chi-square test (2-sided). ^3^ANOVA (2-sided)

### Risk factors for uncontrolled HbA_1c_

The results of logistic regression analyses of glycemic control level in the two groups are presented in Table 3 − 1 and Table 4 − 2, respectively. In the Discont group, the factors of ‘age at resumption’ (OR: 1.05, 95 % CI: 1.01 to 1.09, *p* = 0.016) and ‘current smoking habit’ (OR: 3.59, 95 % CI: 1.29 to 9.99, *p* = 0.014) increased the risk for uncontrolled HbA_1c_, whereas in the Cont group ‘familial history of diabetes’ (OR: 0.42, 95 % CI: 0.19 to 0.88, *p* = 0.022) was associated with decreased risk. The MMAS-8 score representing the level of medication adherence was associated with the risk of uncontrolled HbA_1c_ in the Cont (OR: 0.81, 95 % CI: 0.66 to 0.99, *p* = 0.037) but not in the Discont group. The number of medications were not associated with glycemic control level in both the Cont and the Discont groups.

**Table 3:** − 1 Risk factors for uncontrolled HbA_1c_: Discontinuation group (*n* = 148)

Variable	Odds ratio	95 % CI	*p* value
Age (yr)	1.01	(0.98, 1.06)	0.410
Age at resumption (yr)	1.05	(1.01, 1.09)	0.016
Insulin use	0.40	(0.13, 1.22)	0.108
Drug-related side effects	0.42	(0.17, 1.08)	0.071
Familial history of diabetes	0.54	(0.25, 1.14)	0.106
Current smoking habit	3.59	(1.29, 9.99)	0.014

Dependent variable: controlled (average HbA_1c_ below the assumed target) or uncontrolled (average HbA_1c_ not below the assumed target); odds ratio: uncontrolled versus controlled.

**Table 4:** − 2 Risk factors for uncontrolled HbA_1c_: Continuation group (*n* = 146)

Variable	Odds ratio	95 % CI	*p* value
Complications	0.34	(0.09, 1.28)	0.113
Insulin use	2.28	(0.87, 5.96)	0.092
Hypoglycaemia	2.32	(0.94, 5.74)	0.069
Familial history of diabetes	0.42	(0.19, 0.88)	0.022
MMAS	0.81	(0.66, 099)	0.037

Dependent variable: controlled (average HbA_1c_ did not exceed the assumed target) or uncontrolled (average HbA_1c_ exceeded the assumed target); odds ratio: uncontrolled versus controlled.

### Reasons for discontinuation of pharmacotherapy

All 148 participants in the Discont group answered the question ‘Why did you stop pharmacotherapy?’ Choice- as well as category-wise response rates are shown in Table 5. In choice-wise, the three most frequently given reasons for discontinuation were ‘I was busy with work [family matters]’ (32 %), ‘I was burdened by medical costs’ (22 %), and ‘I was in good shape’ (20 %). In category-wise, social condition (44 %), disease perception (35 %) and relationship with physicians (18 %) were quantified and these were also consolidated as self-judgement (74 %). On the other hand, those who discontinued pharmacotherapy on their physicians’ advice accounted for only 18 %. Approximately three-fourths of the respondents terminated therapy on their own decision for reasons categorized as social conditions, disease perception, and relationship with physicians.

**Table 5:**
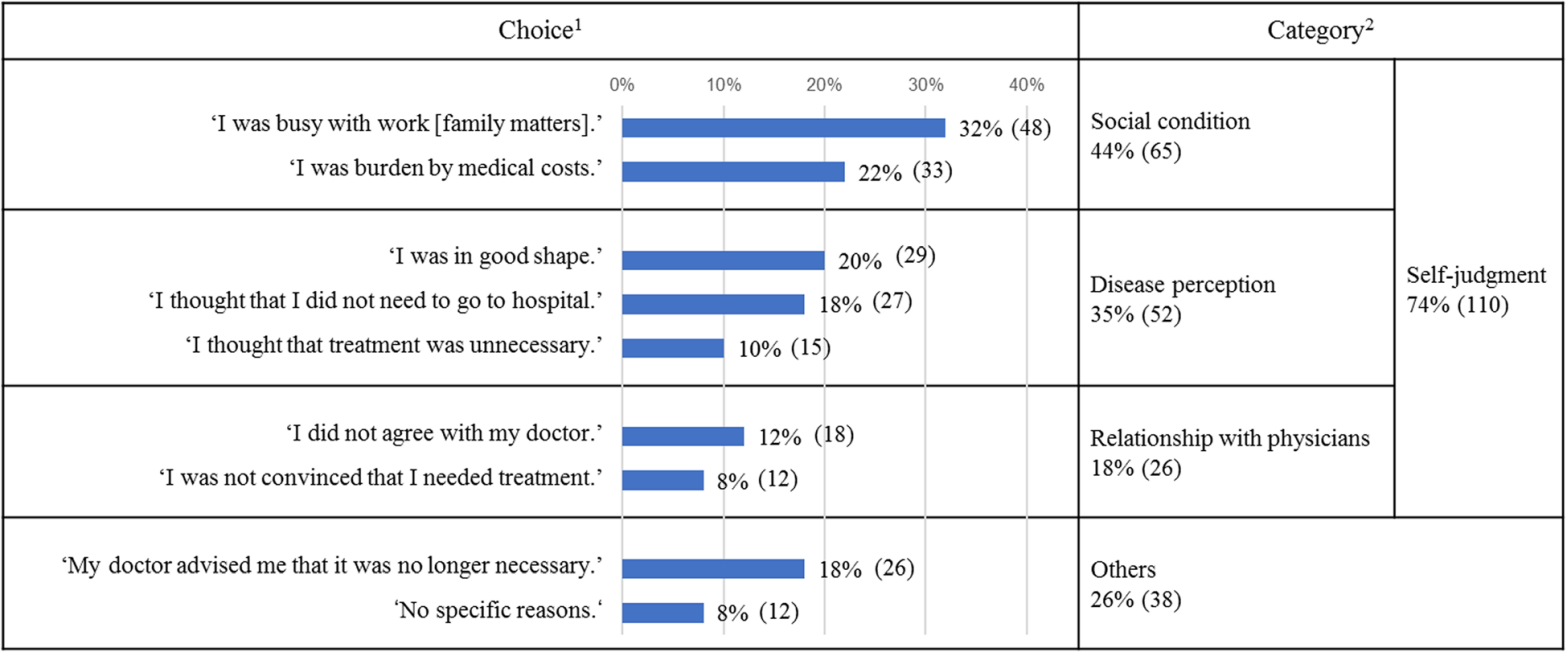
Reasons for discontinuation (*n* = 148). Question: ‘Why did you stop pharmacotherapy? Please choose one or more answers among the following options.’

^1^Participants were allowed to choose multiple choices as needed. ^2^Overlapped counts were eliminated when consolidated.

### Consequences for resumption of pharmacotherapy

One hundred forty participants in the Discont group provided valid answers to the question ‘What triggered your resumption of pharmacotherapy?’ The number of cases and percentages of defined categories are presented in Table 6. Five categories were identified as triggers or factors involved in resuming pharmacotherapy: ‘notified worsening of HbA_1c_’ (39 %), ‘recognized subjective symptoms or complications’ (29 %), ‘incidentally’ (14 %), ‘spontaneous’ (9 %), and ‘regular follow-up’ (4 %).

**Table 6:** Reasons for resumption (*n* = 140). Question: ‘What triggered your resumption of pharmacotherapy?’

Category	Comment	Cases (%)
Received worsening of HbA_1c_	55 (39)	
Recognized subjective symptoms or complications	41 (29)	
Incidentally	I needed to visit hospital for another disease [injury].’	19 (14)
Spontaneous	12 (9)	
Regular follow-up	6 (4)	
Other		7 (5)
Total		140 (100)

## Discussion

No obvious relationship was observed between medication adherence and glycemic control in the Discont group, although it was demonstrated that better adherence is relevant to better glycemic control in the Cont group. While a cross-sectional study like this one does not confirm causality, many other longitudinal studies found that medication adherence contributes to the control of blood glucose levels [18–20]. Those who discontinued pharmacotherapy in the past would not be the same as those who discontinued in light of the impact of medication to the blood glucose. Participants with diabetes in the Discont group tended to be less medication adherent compared with those in the Cont group as seen in the difference of the MMAS-8 score distribution. It means that participants in the Discont group do not take medication as good as those who in the Cont group. Also, those who in the Discont group were not so convinced and accepted to take medication as those who in the Cont group, since ‘adherence’ is conceptually grounded on agreement with recommendation from medical professionals or care providers [21]. As presented in the reasons for resumption of pharmacotherapy in the Discont group, recognizing subjective symptoms or being notified worsening HbA_1c_, have influenced to change their behaviour. However, the degree of understanding in true seriousness of diabetes as the risk of causing micro- and macro-vascular complications may be different in the two groups.

The proportion of current smokers was similar between the Discont (18 %) and Cont (21 %) groups. However, the risk of uncontrolled HbA_1c_ was 3.56-fold higher among smokers than non-smokers in the Discont group, while the risk did not increase significantly among smokers over non-smokers in the Cont group. Consumption (one pack per day in more than 90 % of the smokers), duration of discontinuation, and duration of smoking history were comparable between the two groups. The mean (± SD) smoking duration in the Discont and Cont groups was 31.5 (± 10.9) and 25.7 (± 10.8) years, respectively (data not shown).

Smoking is known to be associated with elevated HbA_1c_ level [22], incidence of chronic kidney disease [23] and incidence of stroke and cardiovascular disease [24]. Thus, patients with diabetes are strongly advised by physicians-in-charge to quit smoking, as recommended in treatment guidelines [3]. Among the current smokers in the Discont and Cont groups, proportion of the patients categorized as highly adherent in the MMAS-8 score were 23 and 33 %, respectively (data not shown). The assumption could be made that smokers in the Cont group do not follow their physicians’ advice on smoking cessation but adhere to medication protocols. On the other hand, smokers in the Discont group do not comply appropriately with medication protocols and possibly other forms of self-care management like diet or exercise and consequently are less likely to achieve the target HbA_1c_ level.

Familial history of diabetes was found to be different between the Dicont and the Cont groups in terms of relationship with the control of HbA_1c_. In the Cont group, the risk of uncontrolled HbA_1c_ of those who have family member(s) with diabetes were 58 % lower than that of those who don’t. There may be positive effects of having family member(s) with diabetes: e.g. receiving instruction about disease and treatment, sharing ideal diet on a daily basis, having better support and care at home, and so on. Also, they might have faced unfavourable seriousness of diabetic complications and had fears, which they would like to avoid. These factors would encourage them to cope with medication and other self-care management properly. While, in the Discont group, there was no significant relationship between the familial history of diabetes and the level of HbA_1c_ control. Proportion of those who have family member(s) in the Discont group (51 %) was slightly higher than in the Cont (43 %) group. A study revealed that having close relatives with diabetes were more complicated in their explanatory model of disease than those who did not have [25]. It suggested that what they learn from their relatives and their sense of efficacy or fear were interrelated in their making decision of treatment behaviour. The difference between the two groups found in the present study may attribute to individual factors that we did not investigate, e.g. family support in the management of diabetes, employment status, participation in educational programs for diabetic patients, etc.

More than 70 % participants in the Discont group decided to discontinue pharmacotherapy without consulting a medical professional. Moreover, almost half of those cases of discontinuation were associated with ‘disease perception,’ for example, ‘I was in good shape,’ ‘I thought that I did not need to go to hospital,’ and ’I thought that treatment was unnecessary,’ which implies inappropriate understanding of diabetes and its treatment. These responses were shown in some reports in the past [10, 15–17], however in our knowledge no studies have been published on how large patients have inappropriate disease perception. It is the primary basis that patients with diabetes should lower their HbA_1c_ levels even if they do not experience subjective symptoms. This must have been instructed by physicians, pharmacists, or other medical professionals at diagnosis and when diabetic individuals begin receiving medication. Notwithstanding, why did they think this way?

According to Festinger’s cognitive dissonance theory [26], we have an inner driver to hold all our attitudes, behaviour, and beliefs in harmony and avoid disharmony (dissonance). When there is an inconsistency among them, we want to change one or more of them to reduce or eliminate such inconsistency. A typical example is the smokers who want to quit smoking but cannot achieve it. They face an inconsistency between ‘belief that smoking is not good for health’ and ‘behaviour of continued smoking’. In this situation, changing the belief, e.g. ‘smoking may be harmful but *not for me*,’ and ‘smoking is not so damaging because there are many smokers who live long and healthy,’ would be made because it is easier than changing the behaviour. Likewise, those who showed inappropriate disease perception in the Discont group in the above might be in the situation of inconsistency between ‘belief that taking medication every day is necessary for my disease’ and ‘behaviour that missing doses.’ Then, they would change the belief, e.g. ‘I do not need to take medication because I am fine.’

The inappropriate disease perception in the Discont group can be also explained by Kahneman’s theory in the field of behavioural economics [27]. They do not act based on a rational balance of risks and rewards, which is assumed in classical economic theory, but often do make irrational decisions. To execute ideal self-care management of diabetes involves laborious efforts to change their daily routines and personal preferences as well as possibly fear of drug-related adverse reactions. Rewards for these burden and risks that incur in the near-term is limited and true benefits, namely preventing diabetic complications, are postponed to the distant future. In this intertemporal decision making across present and future, they are likely to have ‘cognitive bias’ [28]. Depending on the degree of patience and self-control ability, they instinctively presume future value as small from the present standpoint and then struggle to change behaviour for the purpose of future benefits. As a result, it suppresses changing the behaviour in an objectively ideal direction. More importantly, they are not aware of the bias by themselves. If this is the case for patients, it is understandable for medical professionals to have difficulty to find better solution for effective intervention. According to Avorn [29], ‘Despite a growing number of publications about the psychology of decision making, most medical care is still based on a “rational actor” understanding of how we make decisions.’ Although it is yet fully embedded in clinical practice, various interventional studies considering the cognitive bias have already made [30–32] and further pragmatic research and real-world implementation are expected.

Among the triggers for resuming medication in the Discont, ‘receiving worsening of HbA_1c_ level’ and ‘recognition of subjective symptoms’ accounted for 40 % and 30 %, respectively. Many who discontinued pharmacotherapy did not resume it until they were able to reach an understanding of their state of diabetes. Meanwhile, their disease might have advanced in the absence of pharmacotherapy. In this study, the complication rate in the Discont and Cont groups were 15 % and 10 %, respectively, which was not significant. Further investigations in larger populations are needed to analyse in depth underlying reasons for both discontinuing and resuming treatment among individuals with diabetes.

The first study limitation that should be cited is the potential for recall bias concerning reasons for discontinuing and resuming pharmacotherapy because this was a retrospective study design. It is generally difficult to access those who withdraw from treatment in a prospective study because they disappear from the cohort and only a few can be tracked individually thereafter. The present study design allowed the collection of sufficient data to analyse such patients quantitatively. Second, the patients registered in the research company consisted of 59.6 % in male and 16.0 % in the elderly. Its gender balance was almost comparable to the proportion of the elderly and less than that of the entire Japanese diabetic population. This is primarily because of the nature of an internet-based survey. However, because the purpose was to clarify the critical issue of poor adherence with medication regimens, which is likely to occur more in the non-elderly [6], a survey sample of this study was considered medically valuable.

## Conclusions

Those who discontinued pharmacotherapy were less adherent to medication than those who did not discontinue. Risk factors for glycemic control also differed between those who discontinued and those who did not. More than one-third of participants with diabetes who discontinued pharmacotherapy had inappropriate perceptions of their disease, which medical professionals should be aware of for better interventions.

## Data Availability

The dataset analysed during the present study are available from the corresponding author on reasonable request.

## Abbreviations

MMAS: 8-item version of the Morisky Medication Adherence Scale
HbA_1c_: glycosylated haemoglobin
Discont group: patients with diabetes who discontinued pharmacotherapy
Cont group: patients with diabetes who continued pharmacotherapy
